# Regulation of XPO5 phosphorylation by PP2A in hepatocellular carcinoma

**DOI:** 10.1002/mco2.125

**Published:** 2022-04-15

**Authors:** Jiao Li, Jian‐Kang Zhou, Xiaoyu Mu, Shu Shen, Xiaomin Xu, Yao Luo, Yuxin Luo, Yue Ming, Yuangang Wu, Yong Peng

**Affiliations:** ^1^ Laboratory of Molecular Oncology Frontiers Science Center for Disease‐related Molecular Network State Key Laboratory of Biotherapy and Cancer Center West China Hospital Sichuan University Chengdu China

**Keywords:** hepatocellular carcinoma, microRNA, PP2A, XPO5

## Abstract

Exportin 5 (XPO5) is a shuttle protein that mediates precursor miRNA (pre‐miRNA) export from the nucleus to the cytoplasm, an important step in miRNA maturation. We previously demonstrated that XPO5 was phosphorylated by ERK kinase and subsequently underwent conformation change by the peptidyl‐prolyl isomerase Pin1, leading to the reduced miRNA expression in hepatocellular carcinoma (HCC). Protein phosphorylation modification serves as a reversible regulatory mechanism precisely governed by protein kinases and phosphatases. Here we identified that the phosphatase PP2A catalyzed XPO5 dephosphorylation. PP2A holoenzyme is a ternary complex composed of a catalytic subunit, a scaffold subunit, and a regulatory subunit that determines substrate specificity. In this study, we characterized the involvement of B55β subunit in XPO5 dephosphorylation that favored the distribution of XPO5 into the cytoplasm and promoted miRNA expression, leading to HCC inhibition in vitro and in vivo. Our study demonstrates the regulatory role of B55β‐containing PP2A in miRNA expression and may shed light on HCC pathogenesis.

## Graphical abstract

In this study, we characterized the regulatory role of phosphatase PP2A in miRNA expression in hepatocellular carcinoma. PP2A dephosphorylates XPO5 and modulates the pre‐miRNA transport ability of XPO5, thereby promoting miRNA expression.

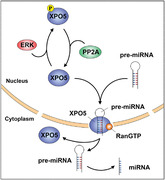

## 1 INTRODUCTION

Hepatocellular carcinoma (HCC) is one of the most prevalent malignancies with high lethality and recurrence worldwide.^1^ The onset and progression of HCC involve multiple external factors combined with altered expression of proteins and noncoding RNAs (ncRNAs). MicroRNAs (miRNAs) represent small ncRNAs of 20–22 nucleotides that tunes gene expression at post‐transcriptional level to maintain homeostasis.^2^ However, the overall expression of miRNAs is downregulated in HCC, which is largely attributed to the defective miRNA biogenesis.^3^, ^4^, ^5^ Therefore, clarifying the molecular mechanisms underlying miRNA biogenesis is essential for understanding HCC pathogenesis.

The canonical miRNA biogenesis initiates with the transcription of miRNA genes mainly by RNA polymerase II into primary transcripts (pri‐miRNAs), which can be subsequently spliced by RNase III Drosha to produce precursor miRNAs (pre‐miRNAs) with a stem loop structure.^6^ With the assistance of RanGTP, exportin 5 (XPO5) transports pre‐miRNAs into the cytoplasm, where they further undergo cleavage by another RNase III Dicer and are loaded onto argonaute (AGO) protein to generate the effector complex called RNA‐induced silencing complex (RISC).^7^

XPO5‐mediated export of pre‐miRNAs is an important step for miRNA maturation.^8^, ^9^, ^10^, ^11^ Overexpression of XPO5 enhances export efficiency and miRNA function.^9^ During cell cycle entry, XPO5 can be promptly induced and serves as a critical molecular hub controlling gene expression via a global elevation of miRNAs.^12^ However, the dysregulation of XPO5 occurs in cancer, which yields profound effect on miRNA expression and aggravates tumorigenesis.^13^, ^14^ In a subset of tumors with microsatellite instability, a genetic defect in XPO5 traps pre‐miRNAs in the nucleus and reduces miRNA processing, whereas reexpression of the wild‐type XPO5 reverses the impaired pre‐miRNA export and the aggressive cellular phenotype, indicating XPO5's tumor‐suppressive property.^13^ Our previous studies showed that ERK kinase‐mediated phosphorylation of XPO5 at proline‐directed serine/threonine sites, coupled with the peptidyl‐prolyl isomerase Pin1‐catalyzed conformation change, impaired the nuclear export of pre‐miRNAs and downregulated miRNA expression during HCC development,^15^, ^16^, ^17^, ^18^ highlighting the important role of phosphorylation in determining XPO5 function. Phosphorylation is a reversible regulatory mechanism precisely controlled by protein kinases and protein phosphatases. Given that serine/threonine phosphatase PP2A can coordinate with Pin1 and catalyze the dephosphorylation of proline‐directed serine/threonine sites on many proteins, such as c‐Myc, Cdc25c, and Tau,^19^, ^20^ we hypothesized that PP2A could be involved in the dephosphorylation of XPO5.

PP2A belongs to the phosphoprotein phosphatase (PPP) superfamily and maintains cellular homoeostasis by counteracting many kinase‐driven signals.^21^ The core enzyme of PP2A is composed of a scaffold subunit (A subunit) and a catalytic subunit (C subunit). To achieve full activity toward particular substrates, the core enzyme further interacts with a variable regulatory subunit (B subunit) to constitute the heterotrimeric PP2A holoenzyme.^22^, ^23^ The B subunit can fall into four unrelated families: B (B55), B’ (B56), B’’ (B72, B130, PR48, and G5PR), and B’’’ (Striatin).^24^ The structure of these subunits varies greatly, allowing for the association of PP2A with diverse substrates. For example, the alpha isoform of B55 is responsible for the dephosphorylation of threonine 308 of Akt,^25^ while the alpha isoform of B56 interacts with c‐Myc and regulates the phosphorylation status of serine 62.^19^ In mammals, both A and C subunits are ubiquitously expressed, while the abundance and subcellular localization of B subunits are tissue and developmental‐stage specific.^26^ Given that distinct B subunits associate with the overlapping binding sites on the A subunit within PP2A core enzyme in a mutually exclusive manner,^27^ the B subunit dictates subcellular compartmentalization and substrate specificity for PP2A.

In this study, we identified that the beta isoform of B55 (B55β)‐containing PP2A is involved in the dephosphorylation of XPO5, thus facilitating XPO5‐mediated miRNA maturation and HCC suppression. Moreover, the expression of B55β was downregulated in HCC tumor samples. Therefore, our findings demonstrate the regulatory role of B55β‐containing PP2A in miRNA expression and HCC pathogenesis, suggesting a novel target for HCC therapy.

## 2 RESULTS

### 2.1 PP2A is responsible for the dephosphorylation of XPO5

**FIGURE 1:**
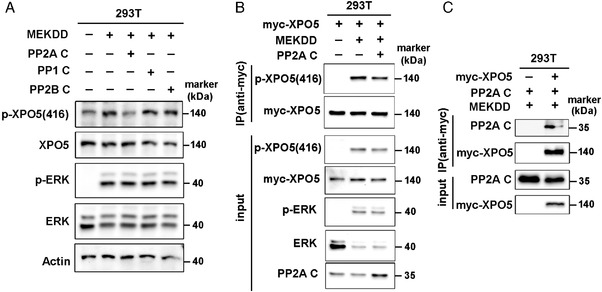
PP2A catalyzes the dephosphorylation of XPO5. (A) HEK‐293T cells were transfected with plasmids expressing MEKDD and different catalytic subunits of serine/threonine phosphatases (PP1, PP2B, and PP2A). The phosphorylated and total XPO5/ERK proteins were detected via Western blot. (B) HEK‐293T cells were transfected with plasmids expressing MEKDD, myc‐XPO5 and C subunits of PP2A and myc‐XPO5 was immunoprecipitated from cell lysates. The immunoprecipitates were immunoblotted with the indicated antibodies. (C) The lysates from HEK‐293T cells co‐transfected with the indicated plasmids were subjected to immunoprecipitation via anti‐myc antibody. The enriched complexes were immunoblotted with the indicated antibodies. MEKDD: constitutively active MEK; p‐XPO5 (416): phosphorylated XPO5 at serine 416.

The PPP family members share high homology in the catalytic subunits.^21^, ^23^ In order to determine which phosphatase is involved in the dephosphorylation of XPO5, we overexpressed constitutively active MEK (MEKDD) in HEK‐293T cells to activate ERK/XPO5 cascade and subsequently transfected these cells with distinct catalytic subunits of serine/threonine phosphatases (PP1, PP2B, and PP2A). The level of phosphorylated XPO5 (p‐XPO5) was decreased upon the overexpression of the C subunit of PP2A (Figure 1A). To further validate the effect of PP2A on XPO5, we transfected HEK‐293T cells with plasmids expressing myc‐tagged XPO5, and immunoprecipitated XPO5 protein with antibody to the myc tag. Clearly, the C subunit of PP2A downregulates p‐XPO5 level (Figure 1B). To test whether XPO5 protein could complex with the PP2A core enzyme, co‐immunoprecipitation was performed and the protein complexes were analyzed. As shown in Figure 1C, XPO5 was specifically recovered with the C subunit of PP2A. These results indicate that PP2A participates in XPO5 dephosphorylation.

### 2.2 The PP2A regulatory subunit B55β mediates the dephosphorylation of XPO5

**FIGURE 2:**
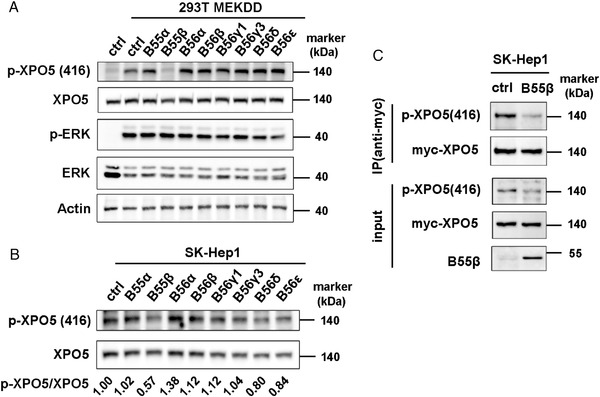
B55β is specifically engaged in XPO5 dephosphorylation. (A) HEK‐293T cells were transfected with plasmids expressing MEKDD and different regulatory subunits. The phosphorylated and total XPO5/ERK proteins were detected via Western blot. (B) SK‐Hep1 cells were transfected with plasmids expressing different regulatory subunits. The phosphorylated and total XPO5 proteins were detected via Western blot and the protein bands were quantified by ImageJ software. Comparison of the intensity of p‐XPO5 bands (using total XPO5 as loading control) was indicated as fold change relative to ctrl set. (C) SK‐Hep1 cells were co‐transfected with the indicated plasmids and myc‐XPO5 was immunoprecipitated from cell lysates. The immunoprecipitates were immunoblotted with the indicated antibodies.

Considering that the B subunit determines the specificity of PP2A substrate, we next tended to identify the B subunit specific for XPO5 dephosphorylation. HEK‐293T cells were transfected with the available B subunits in hand and p‐XPO5 level was measured. As indicated in Figure 2A, B55β subunit particularly decreased p‐XPO5, suggesting the involvement of B55β in XPO5 dephosphorylation. For further validation in HCC, we also screened these regulatory subunits in HCC SK‐Hep1 cells. Consistently, overexpression of B55β promoted the dephosphorylation of XPO5 (Figure 2B). Moreover, when XPO5 was immunoprecipitated in SK‐Hep1 cells following the transfection of B55β, we observed a marked reduction of p‐XPO5 (Figure 2C), supporting the engagement of B55β regulatory subunit in XPO5 dephosphorylation.

### 2.3 B55β reduces the proliferation, migration, and invasion abilities of HCC cells

**FIGURE 3:**
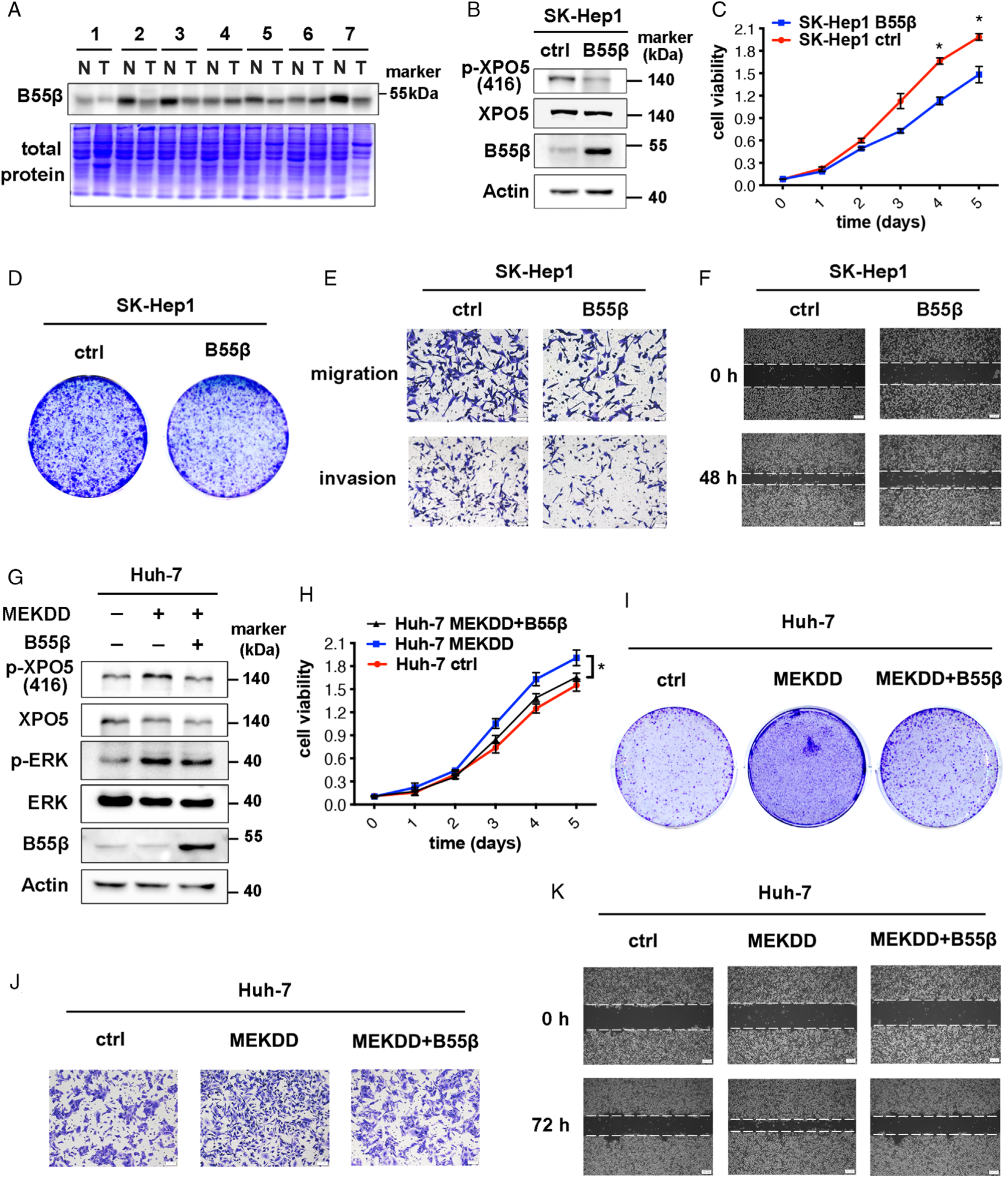
B55β inhibits growth, migration and invasion abilities of HCC cells. (A) The protein lysates of human HCC and adjacent normal tissues were subjected to SDS‐PAGE and then stained with coomassie blue or immunoblotted with anti‐B55β antibody. (B) B55β level was determined by Western blot in SK‐Hep1 stable cell lines with or without B55β overexpression. (C–F) MTT assays (C), cloning formation (D), transwell migration and invasion assays (E), and wound healing (F) for SK‐Hep1 B55β overexpression and ctrl cells. (G) B55β level was detected by Western blot in Huh‐7 stable cell lines with or without MEKDD/B55β overexpression. (H‐K) MTT assays (H), cloning formation (I), transwell migration assays (J), and wound healing (K) for Huh‐7 stable cell lines with or without MEKDD/B55β overexpression. Data are shown as the means ± SD. *p < 0.05.

Compelling evidence highlights that PP2A is functionally inactivated in cancers, typically through loss of heterozygosity and/or aberrant expression of PP2A subunits.^28^ Previous studies reported that the B55β‐coding gene, *PPP2R2B*, was epigenetically silenced due to the upregulation of the histone methyltransferase EZH2 in HCC.^29^ To verify the expression of B55β in HCC, paired tumorous samples and their adjacent normal tissues were collected and analyzed. The results showed that B55β was dramatically reduced in HCC tissues (Figure 3A), suggesting its tumor‐suppressive role in HCC progression. Because SK‐Hep1 and Huh‐7 HCC cell lines show the highest and the lowest p‐ERK/p‐XPO5 levels among the HCC cell lines we examined, respectively,^16^ they were chosen to investigate B55β function. Based on the decreased expression of B55β in HCC, we established the stable cell line overexpressing B55β and scramble vector in SK‐Hep1 cells (SK‐Hep1 B55β and SK‐Hep1 ctrl). SK‐Hep1 B55β cells exhibited a lower p‐XPO5 level as well as a decreased proliferation ability compared to the control cells (Figure 3B–D). Accordingly, B55β overexpression remarkably suppressed cell migration and invasion in SK‐Hep1 cells (Figure 3E and F).

To further determine the phenotype of B55β in HCC, Huh‐7 stable cell line overexpressing MEKDD (Huh‐7 MEKDD) were established to trigger ERK/XPO5 signaling (Figure 3G), along with the promoted cell proliferation ability (Figure 3H and I). However, subsequent upregulation of B55β decreased p‐XPO5 level and rescued cellular phenotypes (Figure 3G–I), implying that B55β might exert its antitumor activity via antagonizing ERK‐induced XPO5 phosphorylation. Moreover, we observed a reversible transformation of cellular morphology in these transfectants (Supplementary Figure S1), supporting the regulatory role of B55β in cell movement. To test this, transwell migration and wound healing assays were performed. As expected, B55β abrogated ERK‐induced cell migration (Figure 3J and K). Collectively, B55β exerts a tumor‐suppressive role in HCC at least in part by counteracting ERK‐driven signals.

### 2.4 B55β regulates miRNA expression in HCC cells

**FIGURE 4:**
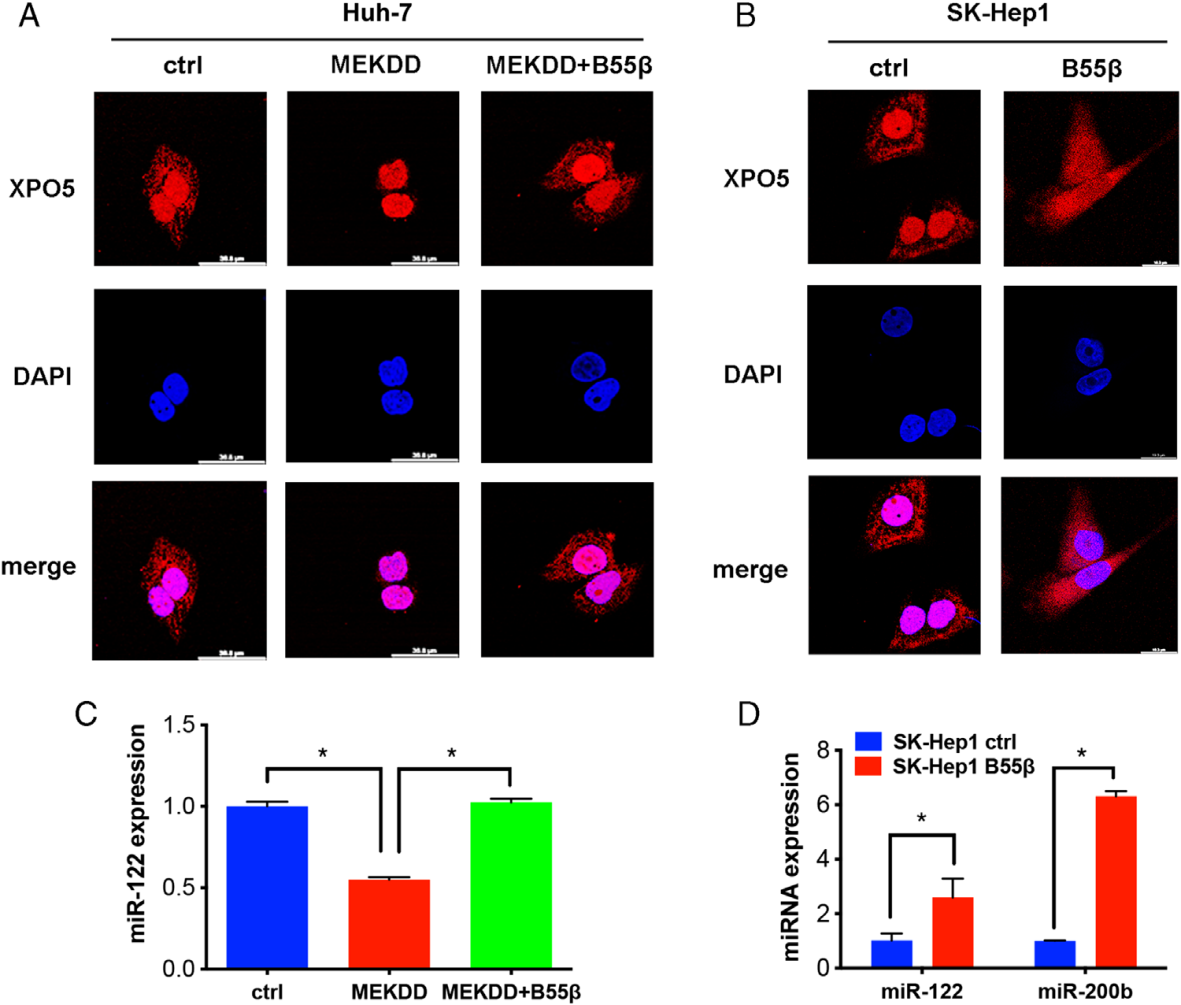
B55β regulates XPO5 distribution and miRNA expression. (A) Immunofluorescence of subcellular localization of XPO5 (Red) in Huh‐7 cells transfected with the indicated plasmids. (B) Immunofluorescence of subcellular localization XPO5 (Red) in SK‐Hep1 Ctrl and B55β overexpression cells. (C) The miR‐122 expression in Huh‐7 cells transfected with the indicated plasmids was determined by qRT‐PCR. (D) The expression of miR‐122 and miR‐200b in SK‐Hep1 ctrl and B55β overexpression cells was examined by qRT‐PCR. Data are shown as the means ± SD. *p < 0.05, **p < 0.01.

Since XPO5 primarily shuttles between the nucleus and the cytoplasm to transport pre‐miRNAs and our previous findings indicated that ERK‐induced XPO5 phosphorylation trapped XPO5 in the nucleus,^15^ we next examined whether B55β‐mediated dephosphorylation could impinge on the subcellular distribution of XPO5. As indicated in Figure 4A and Supplementary Figure S2A, XPO5 was spread in both the nucleus and the cytoplasm of Huh‐7 cells with low XPO5 phosphorylation. Increased XPO5 phosphorylation by ERK activation kept XPO5 in the nucleus, whereas B55β overexpression recovered the cytoplasmic localization of XPO5 (Figure 4A and Supplementary Figure S2A). Analogously, SK‐Hep1 B55β cells with lower p‐XPO5 showed more cytoplasmic distribution of XPO5 when compared with the control cells (Figure 4B and Supplementary Figure S2B). These findings indicate the involvement of B55β in modulating the cellular distribution of XPO5.

Given that XPO5‐mediated nuclear export is important for miRNA maturation, we explored whether B55β‐catalyzed XPO5 dephosphorylation affected miRNA expression. We previously demonstrated that ERK‐triggered XPO5 phosphorylation, coupled with subsequent conformation change, hindered the expression of miR‐122 and miR‐200b in HCC.^15^, ^16^ miR‐122 is a hepatocyte‐specific miRNA accounting for about 70% of total liver miRNAs and exerts a dominant role in liver homeostasis and hepatocarcinogenesis.^30^, ^31^, ^32^ Huh‐7 cells resemble normal hepatocytes in high abundance of miR‐122.^33^ In accord with the alternation in XPO5 compartmentalization (Figure 4A), ERK‐triggered XPO5 phosphorylation impeded miR‐122 expression, which was rescued by B55β augmentation (Figure 4C). The similar results were observed in SK‐Hep1 cells (Figure 4D). Additionally, miR‐200b, a frequently downregulated tumor suppressor in a variety of tumors, was also elevated by B55β‐induced XPO5 dephosphorylation (Figure 4D). Taken together, the expression of these tumor‐suppressive miRNAs is negatively correlated with the level of XPO5 phosphorylation, and the regulatory B55β subunit could antagonize the effect of ERK kinase on XPO5, thereby promoting the expression of these miRNAs.

### 2.5 B55β inhibits cell proliferation and migration through modulating miRNA expression

**FIGURE 5:**
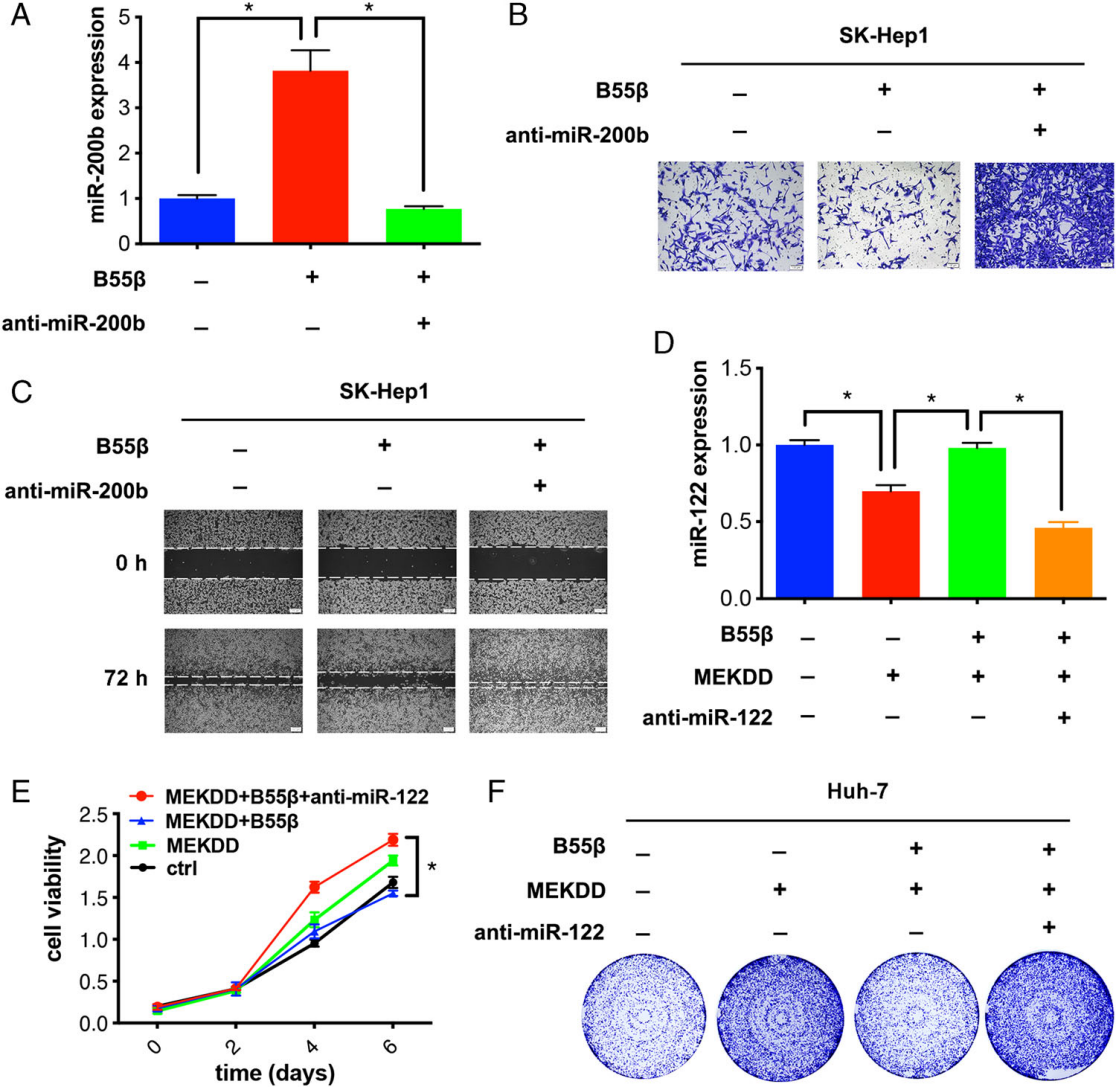
B55β‐induced HCC inhibition is partially attributed to the regulation of miRNA expression. (A) SK‐Hep1 ctrl and B55β overexpression cells were transfected with or without anti‐miR‐200b, followed by the examination of miR‐200b expression via qRT‐PCR. (B) Transwell migration assays and (C) wound healing assays for SK‐Hep1 stable cell lines transfected with or anti‐miR‐200b. (D) miR‐122 expression was detected by qRT‐PCR in Huh‐7 cells transfected with or without anti‐miR‐122. (E) MTT assays and (F) cloning formation assays for Huh‐7 stable cell lines transfected with or without anti‐miR‐122. Data are shown as the means ± SD. *p < 0.05.

Based on the above findings that the regulatory B55β subunit modulates miRNA expression, we further investigated whether B55β exerted its biological significance through affecting these miRNAs. miR‐200b is illustrated to inhibit tumor metastasis by regulating epithelial‐mesenchymal transition (EMT).^34^, ^35^ To provide evidence for the engagement of miR‐200b in B55β‐mediated anti‐HCC program, SK‐Hep1 B55β cells were transfected with anti‐miR‐200b to lower its expression (Figure 5A). As indicated in Figure 5B and C, B55β‐mediated suppression of cell migration was reversed by the subsequent inhibition of miR‐200b, indicating that the antimigratory activity of B55β is achieved in part by upregulating miR‐200b. Similarly, the antiproliferative effect of B55β was weakened upon the inhibition of miR‐122 expression (Figure 5D–F), implying the active involvement of miR‐122 in B55β‐mediated regulatory network. Therefore, B55β‐mediated HCC inhibition is at least in part through elevating these tumor‐suppressive miRNAs.

### 2.6 B55β suppresses HCC development in vivo

**FIGURE 6:**
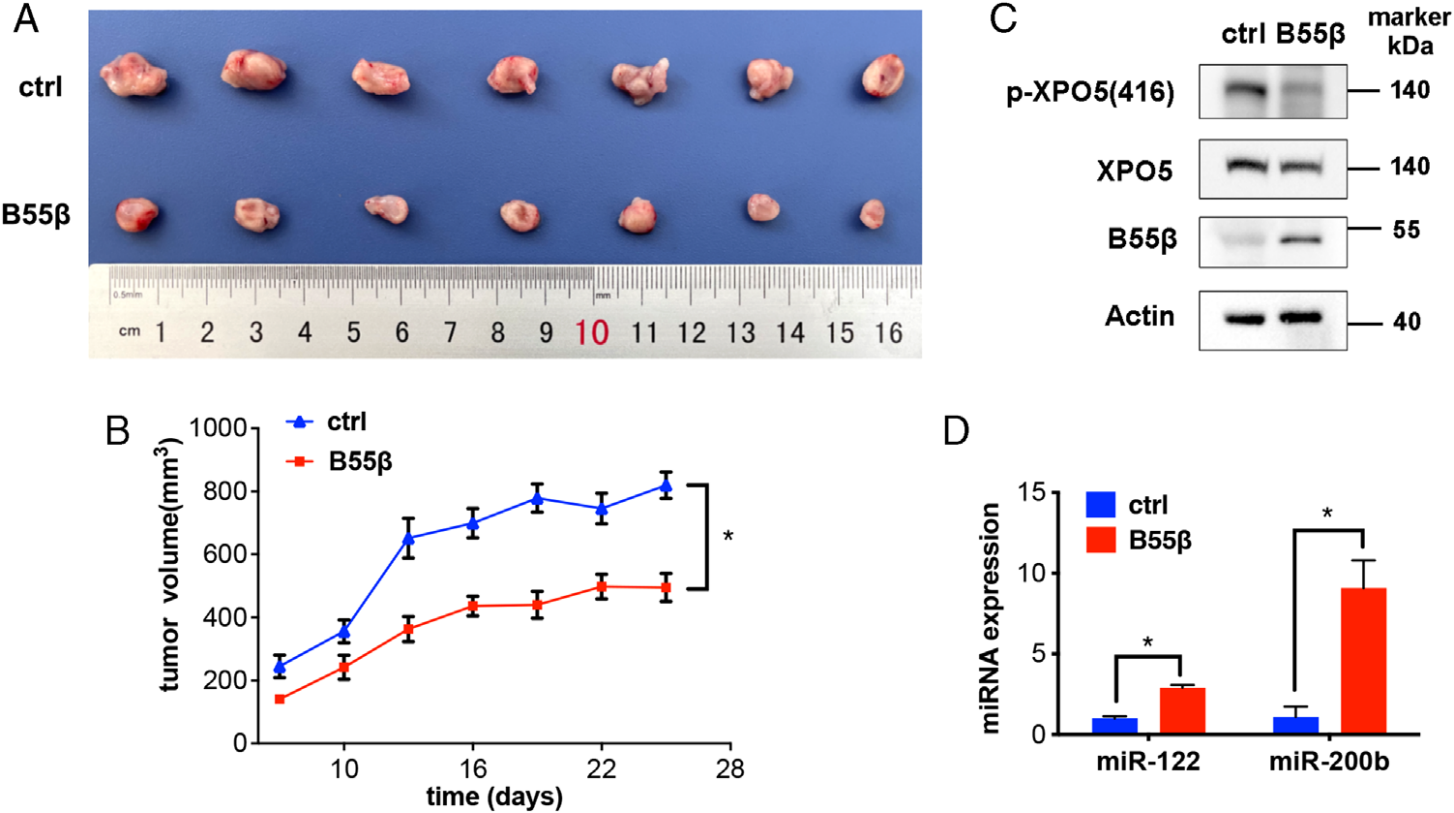
B55β represses HCC progression via tuning miRNA expression in vivo. (A) Photograph and (B) tumor growth curve of SK‐Hep1 ctrl and B55β overexpression xenografts in nude mice. (C) Phosphorylated and total XPO5 as well as B55β in xenografts was detected by Western blot. (D) miRNA expression in xenografts was measured by qRT‐PCR. Data are shown as the means ± SD. *p < 0.05, **p < 0.01.

We next evaluated the in vivo function of B55β in the HCC xenograft model. In line with the above findings, B55β significantly inhibited tumor growth when compared with control set (Figure 6A and B). Importantly, p‐XPO5 was downregulated, whereas miR‐122 and miR‐200b were elevated in SK‐Hep1 B55β tumor (Figure 6C and D), indicating that B55β exerts in vivo anti‐HCC role through dephosphorylating XPO5 and promoting some miRNA expression.

## 3 DISCUSSION

**FIGURE 7:**
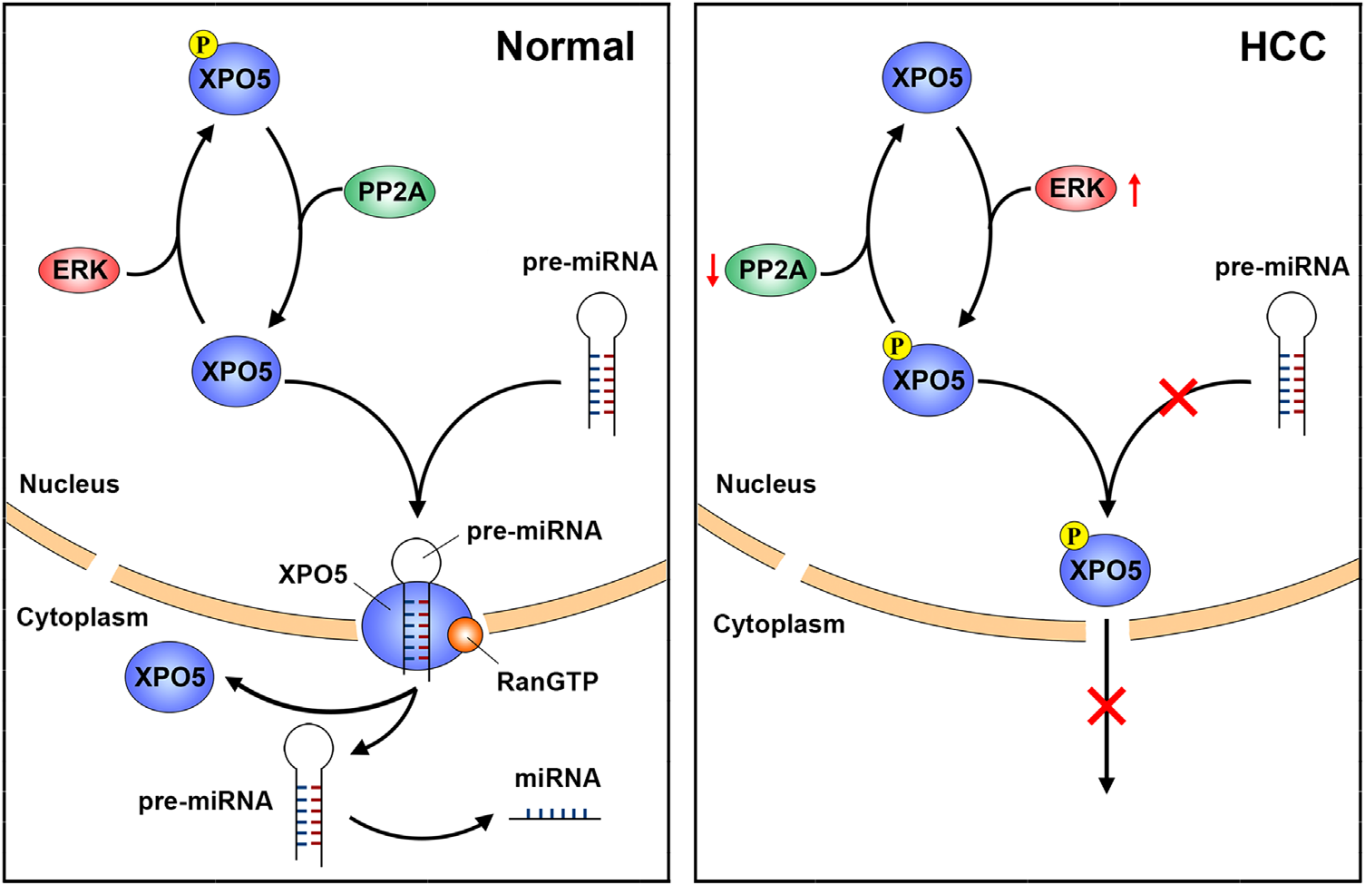
Schematic diagram of how PP2A modulates miRNA expression. In normal conditions, phosphatase PP2A dephosphorylates XPO5 and modulating the transport ability of XPO5, thus promoting miRNA expression. In HCC, PP2A downregulation coupled with ERK activation favors XPO5 phosphorylation, leading to the compromised miRNA expression.

The spatial and temporal expression of miRNAs are key for cell lineage decisions and tissue homeostasis, while the overall downregulation of miRNA is observed in HCC and causally involved in tumor development.^3^, ^4^, ^5^ Increasing evidence supports the defect in miRNA biogenesis drives miRNA dysregulation and subsequent oncogenic transformation of various cell types.^4^, ^5^ Our previous work showed that ERK‐induced phosphorylation of XPO5, followed by Pin1‐mediated isomerization, hindered miRNA biogenesis in HCC.^15^, ^16^, ^17^, ^18^ However, little is informed about the process of XPO5 dephosphorylation. In this study, we revealed that the serine/threonine phosphatase PP2A specifically catalyzed the dephosphorylation of XPO5, while this modulatory mechanism was impaired due to PP2A downregulation in HCC (Figure 7). Further augmentation of PP2A restored the cytoplasmic distribution of XPO5 and promoted the expression of several tumor‐suppressive miRNAs. Thus, our study provides new insight into miRNA dysregulation in HCC.

The PP2A holoenzyme is a ternary complex consisting of a catalytic subunit, a scaffold subunit, as well as a regulatory subunit responsible for recruiting substrates. Among the available PP2A regulatory subunits in hand, we identified that the B55β subunit remarkably reduced the level of p‐XPO5 in both HEK‐293T and HCC cell lines (Figure 2A and B), and further confirmed the involvement of B55β in XPO5 dephosphorylation via immunoprecipitation of XPO5 upon B55 augmentation and examination of the phosphorylated status of the recovered protein (Figure 2C). In view of the diversity of the PP2A regulatory subunit family, we can't rule out the possibility that other regulatory subunits are also engaged in the regulation of XPO5 phosphorylation.

PP2A primarily functions as a tumor suppressor through tuning the phosphorylation status and the function of important cancer‐associated proteins, such as c‐Myc and p53.^19^, ^36^, ^37^ Our study identified XPO5 as a potential substrate for B55β‐containing PP2A and further clarified that B55β‐containing PP2A can inhibit HCC progression by promoting XPO5‐dependent maturation of several tumor‐suppressive miRNAs, providing another potential mechanism to interpret the anti‐tumor role of PP2A. Cancer cells employ various strategies to evade PP2A‐mediated tumor suppression, such as dysregulation of PP2A subunits and its binding partners, or loss of phosphatase activity.^38^, ^39^, ^40^, ^41^ For example, *PPP2R2A*, the α isoform of B55 regulatory subunit family, is frequently deleted in luminal B breast cancers, which adds a significant pathophysiological feature to this cancer subtype.^39^ In this study, we examined expression of B55β subunit in paired HCC and adjacent normal samples and showed the general downregulation of B55β in HCC tissues (Figure 3A), supporting it as a potential target for HCC therapy. Currently, pharmacological strategies for targeting PP2A in human cancer are developed. For example, small molecules are designed to selectively promote the assembly of the holoenzyme with specific subunits.^42^, ^43^, ^44^ These findings instill promise for restoring the function of B55β‐containg PP2A in HCC in a selective manner.

Our findings found that PP2A‐mediated dephosphorylation of XPO5 restored the biogenesis of several important tumor‐suppressive miRNAs (e.g., miR‐122 and miR‐200b) via antagonizing ERK kinase (Figure 4). MiR‐122, the most abundant miRNA in normal liver, is drastically downregulated in HCC.^45^ Deletion of the *miR‐122* gene leads to hepatocarcinogenesis in mice.^31^, ^32^ MiR‐200b is another key tumor‐suppressive miRNA that inhibits tumor metastasis through regulating various proteins, such as ZEB and moesin.^34^, ^35^, ^46^ However, it is clear that not all miRNA is governed by PP2A/XPO5 axis. In addition to the canonical miRNA biogenesis, recent studies have characterized the alternative pathways for miRNA processing.^6^, ^47^ During cellular quiescence, a subset of pri‐miRNAs with a 2,2,7‐trimethylguanosine (TMG)‐cap is induced and processed in a XPO5‐independent but XPO1‐dependant manner.^47^ Therefore, miRNA biogenesis is much more complex than previously supposed and further work is needed to illustrate the detailed network.

## 4 MATERIALS AND METHODS

### 4.1 Reagents

Anti‐XPO5, anti‐actin, anti‐PP2A catalytic subunit, anti‐p‐ERK, anti‐ERK, and anti‐myc tag antibodies for Western blot were from Cell Signaling Technology (Danvers, MA, USA). Anti‐B55β antibody for Western blot was purchased from Proteintech (Wuhan, China). Anti‐XPO5 antibody for immunofluorescence was from Novus Biologicals (USA). Antibodies to the Ser416 phosphorylation site of XPO5 were generated in collaboration with Lifetein LLC. Anti‐c‐Myc agarose beads were from Sigma‐Aldrich (St. Louis, MO, USA). Anti‐miRNA oligos were purchased from GenePharma Company.

### 4.2 Plasmids

The plasmids B55α (#13804), B55β (#16181), B56α (#14532), B56β (#14533), B56γ1 (#14534), B56γ3 (#14535), B56δ (#14536), and B56ε (14537) were bought from Addgene. The full length of B55β (#16181, Addgene) cDNA was subcloned into the pCDH‐CMV‐MCS‐EF1‐copGFP vector.

### 4.3 Cell culture

SK‐Hep1, Huh‐7, and HEK‐293T cells were cultured in DMEM medium with 10% fetal bovine serum at 37°C in a 5% CO_2_ incubator.

### 4.4 Cell transfection

For transient expression, cells were transfected with plasmids using Lipofectamine 2000 transfection reagent. Briefly, cells were seeded into 6‐well plates at a density of 3 × 10^5^ cells per well and incubated overnight prior to transfection. Plasmids and Lipofectamine 2000 were diluted separately in Opti‐MEM medium (Invitrogen) and left for 5 min at room temperature. The diluted DNA and Lipofectamine 2000 were then gently mixed together at a ratio of 1 μg DNA/2.5 μl Lipofectamine 2000 and incubated for 20 min at room temperature. Finally, the mixture was added to cells and incubated for a certain time before performing subsequent experiments. The medium was changed after 6 h. For lentivirus preparation, HEK‐293T cells were co‐transfected with pCDH‐CMV‐MCS‐EF1‐copGFP plasmids expressing B55β, the packaging plasmid pCMV‐dR8.2 dvpr and the envelope plasmid pCMV‐VSVG (System Bioscience). SK‐Hep1 and Huh‐7 cells were infected with the resultant lentivirus and stable cell lines were obtained after GFP sorting.

### 4.5 Clinical samples

Primary HCC tumor tissues and the adjacent normal tissues were obtained from West China Hospital with written informed consent for research purpose. All procedures were approved by the Ethics Committee of West China Hospital of Sichuan University. For total protein preparation, the patient tissues were cut up and grounded into powder in liquid nitrogen. Subsequently, RIPA buffer was added and sonicated, followed by centrifugation at 12,000 rpm for 15 min at 4°C. The resultant supernatants were collected for Western blot analysis.

### 4.6 Western blot

Total proteins were extracted with RIPA buffer (25 mM Tris‐HCl, pH 7.5, 0.1% SDS, 1% Nonidet P‐40, 1% sodium deoxycholate, 150 mM NaCl) supplemented with phosphatase inhibitor and protease inhibitor cocktail and quantified with the BCA assay. Equal amounts of proteins were added to each well of SDS‐PAGE gels and transferred to PVDF membranes (Millipore). After blocking with 5% nonfat milk or BSA for 1 h at room temperature, membranes were incubated with primary antibody at 4°C overnight and secondary antibody at room temperature for 1 h. The chemiluminescence signals were detected using SuperSignal West Dura Extended Duration Substrate (Thermo Scientific).

### 4.7 MTT assay

Cells were seeded into 96‐well plates at a density of 3 × 10^3^ cells per well and cultured for a certain time. At the indicated time points, 10 μl of MTT (5 mg/ml, Sigma) was added into each well and incubated at 37°C for 3 h. After the supernatant was removed, the resultant purple formazan crystals were dissolved in DMSO. The absorbance was measured at a wavelength of 570nm by UV spectrophotometer (Thermal Fisher).

### 4.8 Cloning formation assay

Cells were seeded into 6‐well plates at a density of 3 × 10^3^ cells per well and cultured for several days. The plates were then fixed by 4% paraformaldehyde (PFA) for 20 min and stained with 1% crystal violet for 15 min.

### 4.9 Transwell migration and invasion assay

Transwell chambers (8.0 μm) with or without Matrigel coating were used for cell migration and invasion assays. For migration assay, 1 × 10^5^ cells were suspended in 200 μl of serum‐free medium and seeded into the top chambers in the 24‐well plate, while medium with 10% FBS was added into the bottom chambers. For invasion assay, the chamber was precoated with Matrigel (BD Biosciences) and 2 × 10^5^ cells were seeded in each chamber. After 24 h incubation, cells in the top chambers were fixed with 4% PFA for 20 min and stained with 1% crystal violet for 15 min.

### 4.10 Wound healing assays

For wound healing assays, cells were cultured in 6‐well plates to full confluency. The sterilized pipet tips were used to scratch the monolayer cells. After washing three times with PBS, cells were cultured in DMEM medium with 0.5% FBS. Images were taken using the microscope after 48 or 72 h.

### 4.11 Immunoprecipitation assay

The immunoprecipitation assay was performed as previously described.^16^ Briefly, cells were co‐transfected with plasmids expressing myc‐XPO5 and B55β. After 48 h of transfection, cells were collected and lysed with cold lysis buffer (0.1% NP‐40, 100 mM NaCl, 20 mM Tris‐HCl, pH 7.5, 0.5 mM EDTA, and protease and phosphatase inhibitor cocktail). After incubation on ice for 15 min, cells were homogenized for 30 strokes. The lysates were then centrifugated at 2000 × *g* for 15 min and subjected to immunoprecipitation with anti‐myc agarose beads (Sigma). Immunoprecipitates were washed three times with washing buffer (0.5% NP‐40, 150 mM NaCl, 0.5 mM EDTA, 20 mM Tris‐HCl, pH 7.5). The bound proteins were eluted with SDS‐PAGE loading buffer for Western blot.

### 4.12 Immunofluorescence analysis

Huh‐7 or SK‐Hep1 stable transfectants were seeded on the glass cover slides and incubated overnight. Then cells were fixed with 4% PFA, permeabilized with 0.5% Triton X‐100, blocked with 7% BSA, and incubated with anti‐XPO5 antibody at 4°C overnight. After washing with PBS three times, cells were incubated with Alexa Fluor 647 dye‐conjugated secondary antibodies at room temperature for 1 h and stained with DAPI solution for 10 min at room temperature. Confocal fluorescence images were acquired using Leica STELLARIS 5 confocal spectral microscope.

### 4.13 miRNA quantification

TRIzol reagent (Invitrogen) was used to extract total RNAs from cells, and M‐MLV Reverse Transcriptase Kit (Invitrogen) was used to generate cDNAs according to the manufacturer's instructions. qRT‐PCR was performed to examine the levels of mature miRNAs by SYBR Green Master Mix Kit (Applied Biosystems) and QuantStudio™ 6 Flex Real‐Time PCR System (Applied Biosystems). U6 was used as the internal control. Primers specific for miRNA quantification were ordered from GenePharma.

### 4.14 Animal experiment

All the procedures in mice experiments were approved by the Ethics Committee of West China Hospital of Sichuan University. Mice were randomly assigned to experimental groups. SK‐Hep1 stable cell lines were collected and suspended at 5 × 10^6^/ml in saline solution with 50% Matrigel (Corning). Then 100 μl of cells were subcutaneously injected into BALB/c nude mice. The length and width of xenografts were measured every 3 days with calipers. Tumor volume was calculated using the formula: volume (mm^3^) = *ab* ^2^/2 (*a*, long diameter; *b*, short diameter). Proteins and RNAs in xenografts were extracted for B55β as well as phosphorylated and total XPO5 detection.

### 4.15 Statistical analysis

The experimental data were represented as the mean ± SD of at least three biological replicates. Two‐tailed Student's *t* test was used to determine the statistical significance of parametric data. Statistical tests were analyzed using GraphPad Prism software. *p* Values less than 0.05 was considered statistically significant.

## CONFLICT OF INTEREST

The author Yong Peng is an associate editor of *MedComm* and not involved in the journal's review of, or decisions related to, this manuscript. The authors declare no conflict of interest.

## AUTHOR CONTRIBUTIONS

Jiao Li and Jian‐Kang Zhou performed cell biology, biochemistry, and animal experiments; Xiaoyu Mu helped to construct the plasmids; Shu Shen and Xiaomin Xu provided patient samples; Yao Luo and Yuxin Luo helped to perform immunofluorescence assay; Yue Ming and Yuangang Wu helped to establish xenograft models. Jiao Li, Jian‐Kang Zhou and Yong Peng wrote the manuscript; Yong Peng oversaw the experimental design and data analysis.

## ETHICS APPROVAL

All animal and patient sample studies were approved by the Ethics Committee of West China Hospital of Sichuan University. Written informed consent for research purposes was obtained from the patients.

## Supporting information

Supporting information

## Data Availability

All data are available from the corresponding authors upon request.

## References

[1] Sung H , Ferlay J , Siegel RL , et al. Global cancer statistics 2020: GLOBOCAN estimates of incidence and mortality worldwide for 36 cancers in 185 countries. CA Cancer J Clin. 2021;71(3):209‐249. 33538338 10.3322/caac.21660

[2] Gebert LFR , MacRae IJ . Regulation of microRNA function in animals. Nat Rev Mol Cell Biol. 2019;20(1):21‐37. 30108335 10.1038/s41580-018-0045-7 PMC6546304

[3] Wong CM , Wong CC , Lee JM , et al. Sequential alterations of microRNA expression in hepatocellular carcinoma development and venous metastasis. Hepatology. 2012;55(5):1453‐1461. 22135159 10.1002/hep.25512

[4] Lin S , Gregory RI . MicroRNA biogenesis pathways in cancer. Nat Rev Cancer. 2015;15(6):321‐333. 25998712 10.1038/nrc3932 PMC4859809

[5] Lee EJ , Baek M , Gusev Y , et al. Systematic evaluation of microRNA processing patterns in tissues, cell lines, and tumors. RNA. 2008;14(1):35‐42. 18025253 10.1261/rna.804508 PMC2151027

[6] Treiber T , Treiber N , Meister G . Regulation of microRNA biogenesis and its crosstalk with other cellular pathways. Nat Rev Mol Cell Biol. 2019;20(1):5‐20. 30228348 10.1038/s41580-018-0059-1

[7] Ha M , Kim VN . Regulation of microRNA biogenesis. Nat Rev Mol Cell Biol. 2014;15(8):509‐524. 25027649 10.1038/nrm3838

[8] Okada C , Yamashita E , Lee SJ , et al. A high‐resolution structure of the pre‐microRNA nuclear export machinery. Science. 2009;326(5957):1275‐1279. 19965479 10.1126/science.1178705

[9] Yi R , Doehle BP , Qin Y , et al. Overexpression of exportin 5 enhances RNA interference mediated by short hairpin RNAs and microRNAs. RNA. 2005;11(2):220‐226. 15613540 10.1261/rna.7233305 PMC1370710

[10] Bohnsack MT , Czaplinski K , Gorlich D . Exportin 5 is a RanGTP‐dependent dsRNA‐binding protein that mediates nuclear export of pre‐miRNAs. RNA. 2004;10(2):185‐191. 14730017 10.1261/rna.5167604 PMC1370530

[11] Zeng Y , Cullen BR . Structural requirements for pre‐microRNA binding and nuclear export by exportin 5. Nucleic Acids Res. 2004;32(16):4776‐4785. 15356295 10.1093/nar/gkh824 PMC519115

[12] Iwasaki YW , Kiga K , Kayo H , et al. Global microRNA elevation by inducible exportin 5 regulates cell cycle entry. RNA. 2013;19(4):490‐497. 23431327 10.1261/rna.036608.112 PMC3677259

[13] Melo SA , Moutinho C , Ropero S , et al. A genetic defect in exportin‐5 traps precursor microRNAs in the nucleus of cancer cells. Cancer Cell. 2010;18(4):303‐315. 20951941 10.1016/j.ccr.2010.09.007

[14] Chiosea S , Jelezcova E , Chandran U , et al. Overexpression of Dicer in precursor lesions of lung adenocarcinoma. Cancer Res. 2007;67(5):2345‐2350. 17332367 10.1158/0008-5472.CAN-06-3533

[15] Sun HL , Cui R , Zhou J , et al. ERK activation globally downregulates miRNAs through phosphorylating exportin‐5. Cancer Cell. 2016;30(5):723‐736. 27846390 10.1016/j.ccell.2016.10.001 PMC5127275

[16] Li J , Pu W , Sun HL , et al. Pin1 impairs microRNA biogenesis by mediating conformation change of XPO5 in hepatocellular carcinoma. Cell Death Differ. 2018;25(9):1612‐1624. 29445125 10.1038/s41418-018-0065-z PMC6143530

[17] Pu W , Li J , Zheng Y , et al. Targeting Pin1 by inhibitor API‐1 regulates microRNA biogenesis and suppresses hepatocellular carcinoma development. Hepatology. 2018;68(2):547‐560. 29381806 10.1002/hep.29819

[18] Zheng Y , Pu W , Li J , et al. Discovery of a prenylated flavonol derivative as a Pin1 inhibitor to suppress hepatocellular carcinoma by modulating microRNA biogenesis. Chem Asian J. 2019;14(1):130‐134. 30474357 10.1002/asia.201801461

[19] Yeh E , Cunningham M , Arnold H , et al. A signalling pathway controlling c‐Myc degradation that impacts oncogenic transformation of human cells. Nat Cell Biol. 2004;6(4):308‐318. 15048125 10.1038/ncb1110

[20] Zhou XZ , Kops O , Werner A , et al. Pin1‐dependent prolyl isomerization regulates dephosphorylation of Cdc25C and tau proteins. Mol Cell. 2000;6(4):873‐883. 11090625 10.1016/s1097-2765(05)00083-3

[21] Shi Y . Serine/threonine phosphatases: mechanism through structure. Cell. 2009;139(3):468‐484. 19879837 10.1016/j.cell.2009.10.006

[22] Xu Y , Xing Y , Chen Y , et al. Structure of the protein phosphatase 2A holoenzyme. Cell. 2006;127(6):1239‐1251. 17174897 10.1016/j.cell.2006.11.033

[23] Cho SU , Xu W . Crystal structure of a protein phosphatase 2A heterotrimeric holoenzyme. Nature. 2007;445(7123):53‐57. 17086192 10.1038/nature05351

[24] Perrotti D , Neviani P . Protein phosphatase 2A: a target for anticancer therapy. Lancet Oncol. 2013;14(6):e229‐e238. 23639323 10.1016/S1470-2045(12)70558-2 PMC3913484

[25] Kuo YC , Huang KY , Yang YS , et al. Regulation of phosphorylation of Thr308 of Akt, cell proliferation and survival by the B55alpha regulatory subunit targeting of the protein phosphatase 2A holoenzyme to Akt. J Biol Chem. 2008;283(4):1881‐1892. 10.1074/jbc.M709585200 18042541

[26] Bononi A , Agnoletto C , De Marchi E , et al. Protein kinases and phosphatases in the control of cell fate. Enzyme Res. 2011;2011:329098. 21904669 10.4061/2011/329098 PMC3166778

[27] Janssens V , Longin S , Goris J . PP2A holoenzyme assembly: in cauda venenum (the sting is in the tail). Trends Biochem Sci. 2008;33(3):113‐121. 18291659 10.1016/j.tibs.2007.12.004

[28] O'Connor CM , Perl A , Leonard D , et al. Therapeutic targeting of PP2A. Int J Biochem Cell Biol. 2018;96:182‐193. 29107183 10.1016/j.biocel.2017.10.008 PMC5927617

[29] Cheng SLA , Lau SS , Chen Y , et al. EZH2‐mediated concordant repression of Wnt antagonists promotes beta‐catenin‐dependent hepatocarcinogenesis. Cancer Res. 2011;71(11):4028‐4039. 21512140 10.1158/0008-5472.CAN-10-3342

[30] Jopling C . Liver‐specific microRNA‐122: biogenesis and function. RNA Biol. 2012;9(2):137‐142. 22258222 10.4161/rna.18827 PMC3346312

[31] Hsu SH , Wang B , Kota J , et al. Essential metabolic, anti‐inflammatory, and anti‐tumorigenic functions of miR‐122 in liver. J Clin Invest. 2012;122(8):2871‐2883. 22820288 10.1172/JCI63539 PMC3408748

[32] Tsai WC , Hsu SD , Hsu CS , et al. MicroRNA‐122 plays a critical role in liver homeostasis and hepatocarcinogenesis. J Clin Invest. 2012;122(8):2884‐2897. 22820290 10.1172/JCI63455 PMC3408747

[33] Chang J , Nicolas E , Marks D , et al. MiR‐122, a mammalian liver‐specific microRNA, is processed from hcr mRNA and may downregulate the high affinity cationic amino acid transporter CAT‐1. RNA Biol. 2004;1(2):106‐113. 17179747 10.4161/rna.1.2.1066

[34] Korpal M , Lee ES , Hu G , et al. The miR‐200 family inhibits epithelial‐mesenchymal transition and cancer cell migration by direct targeting of E‐cadherin transcriptional repressors ZEB1 and ZEB2. J Biol Chem. 2008;283(22):14910‐14914. 18411277 10.1074/jbc.C800074200 PMC3258899

[35] Gollavilli PN , Parma B , Siddiqui A , et al. The role of miR‐200b/c in balancing EMT and proliferation revealed by an activity reporter. Oncogene. 2021;40(12):2309‐2322. 33654197 10.1038/s41388-021-01708-6 PMC7994202

[36] Shouse GP , Nobumori Y , Liu X . A B56gamma mutation in lung cancer disrupts the p53‐dependent tumor‐suppressor function of protein phosphatase 2A. Oncogene. 2010;29(27):3933‐3941. 20473327 10.1038/onc.2010.161 PMC2900437

[37] Li HH , Cai X , Shouse GP , et al. A specific PP2A regulatory subunit, B56gamma, mediates DNA damage‐induced dephosphorylation of p53 at Thr55. EMBO J. 2007;26(2):402‐411. 17245430 10.1038/sj.emboj.7601519 PMC1783465

[38] Mumby M . PP2A: unveiling a reluctant tumor suppressor. Cell. 2007;130(1):21‐24. 17632053 10.1016/j.cell.2007.06.034

[39] Curtis C , Shah SP , Chin SF , et al. The genomic and transcriptomic architecture of 2,000 breast tumours reveals novel subgroups. Nature. 2012;486(7403):346‐352. 22522925 10.1038/nature10983 PMC3440846

[40] Neviani P , Santhanam R , Trotta R , et al. The tumor suppressor PP2A is functionally inactivated in blast crisis CML through the inhibitory activity of the BCR/ABL‐regulated SET protein. Cancer Cell. 2005;8(5):355‐368. 16286244 10.1016/j.ccr.2005.10.015

[41] Junttila RM , Puustinen P , Niemelä M , et al. CIP2A inhibits PP2A in human malignancies. Cell. 2007;130(1):51‐62. 17632056 10.1016/j.cell.2007.04.044

[42] Morita K , He S , Nowak RP , et al. Allosteric activators of protein phosphatase 2A display broad antitumor activity mediated by dephosphorylation of MYBL2. Cell. 2020;181(3):702‐715. 32315619 10.1016/j.cell.2020.03.051 PMC7397863

[43] Leonard D , Huang W , Izadmehr S , et al. Selective PP2A enhancement through biased heterotrimer stabilization. Cell. 2020;181(3):688‐701. 32315618 10.1016/j.cell.2020.03.038 PMC7243596

[44] Vervoort SJ , Welsh SA , Devlin JR , et al. The PP2A‐Integrator‐CDK9 axis fine‐tunes transcription and can be targeted therapeutically in cancer. Cell. 2021;184(12):3143‐3162. 34004147 10.1016/j.cell.2021.04.022 PMC8567840

[45] Wong CM , Tsang FH , Ng IO . Non‐coding RNAs in hepatocellular carcinoma: molecular functions and pathological implications. Nat Rev Gastroenterol Hepatol. 2018;15(3):137‐151. 29317776 10.1038/nrgastro.2017.169

[46] Li X , Roslan S , Johnstone CN , et al. MiR‐200 can repress breast cancer metastasis through ZEB1‐independent but moesin‐dependent pathways. Oncogene. 2014;33(31):4077‐4088. 24037528 10.1038/onc.2013.370

[47] Martinez I , Hayes KE , Barr JA , et al. An exportin‐1‐dependent microRNA biogenesis pathway during human cell quiescence. Proc Natl Acad Sci U S A. 2017;114(25):E4961‐E4970. 28584122 10.1073/pnas.1618732114 PMC5488920

