# Supplementary material for: Regulation of XPO5 phosphorylation by PP2A in hepatocellular carcinoma
Source: MedComm (2020). 2022 Apr 15;3(2):e125. doi: 10.1002/mco2.125 (PMC9012160; doi:10.1002/mco2.125)
Supplement: Supplementary file 1 — Supporting information [file MCO2-3-e125-s001.docx]

**Regulation of XPO5 phosphorylation by PP2A in hepatocellular carcinoma**

Jiao Li^1,a^, Jian-kang Zhou^1,a^, Xiaoyu Mu^1^, Shu Shen^1^, Xiaomin Xu^1^, Yao Luo^1^, Yuxin Luo^1^, Yue Ming^1^, Yuangang Wu^1^, and Yong Peng^1,^*

^1^Laboratory of Molecular Oncology, Frontiers Science Center for Disease-related Molecular Network, State Key Laboratory of Biotherapy and Cancer Center, West China Hospital, Sichuan University, Chengdu, 610064 China.

**Running Title**: Regulation of XPO5 phosphorylation by PP2A

***Corresponding author:** Yong Peng

E-mail address: yongpeng@scu.edu.cn (Y. P.)

^a^ These authors contributed equally to this work.

**Supplementary Information**


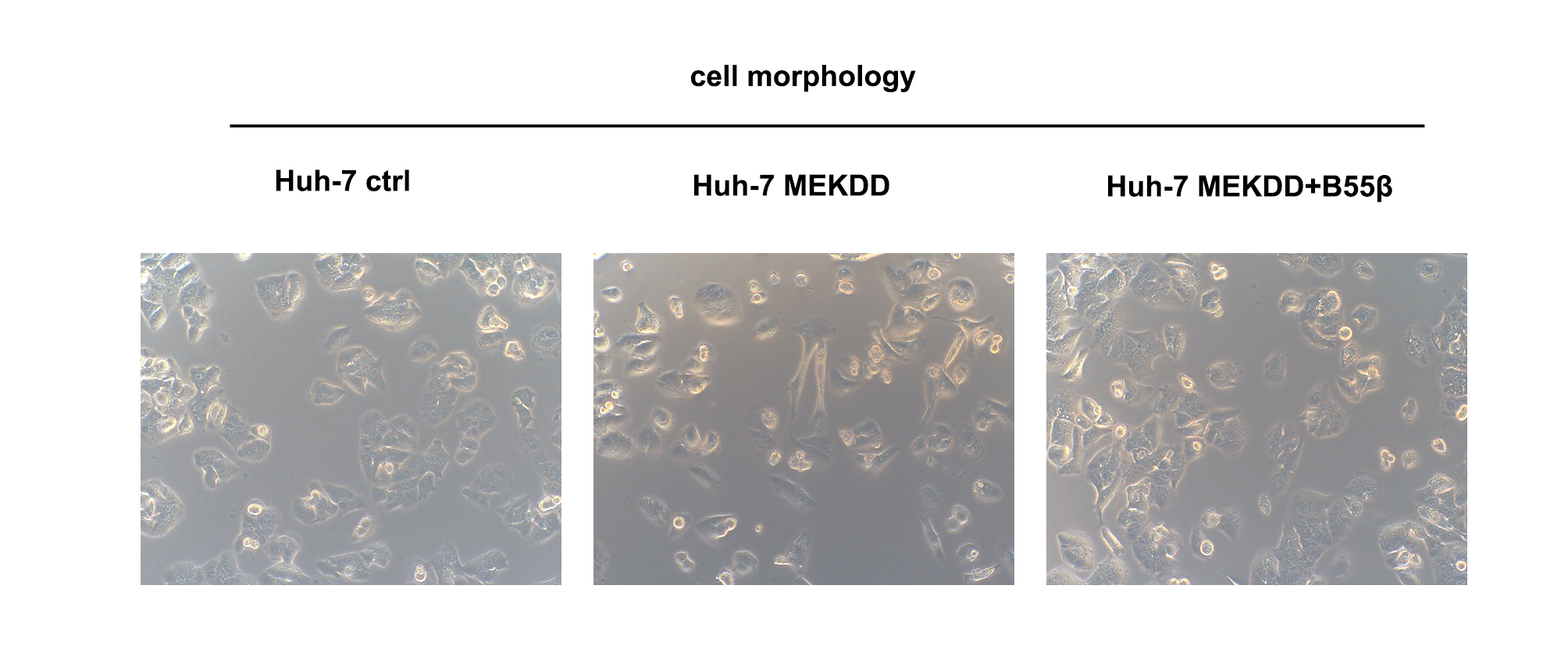


**SUPPLEMENTARY FIGURE 1** The morphology of Huh7 cells transfected with MEKDD and/or B55β plasmids.

**
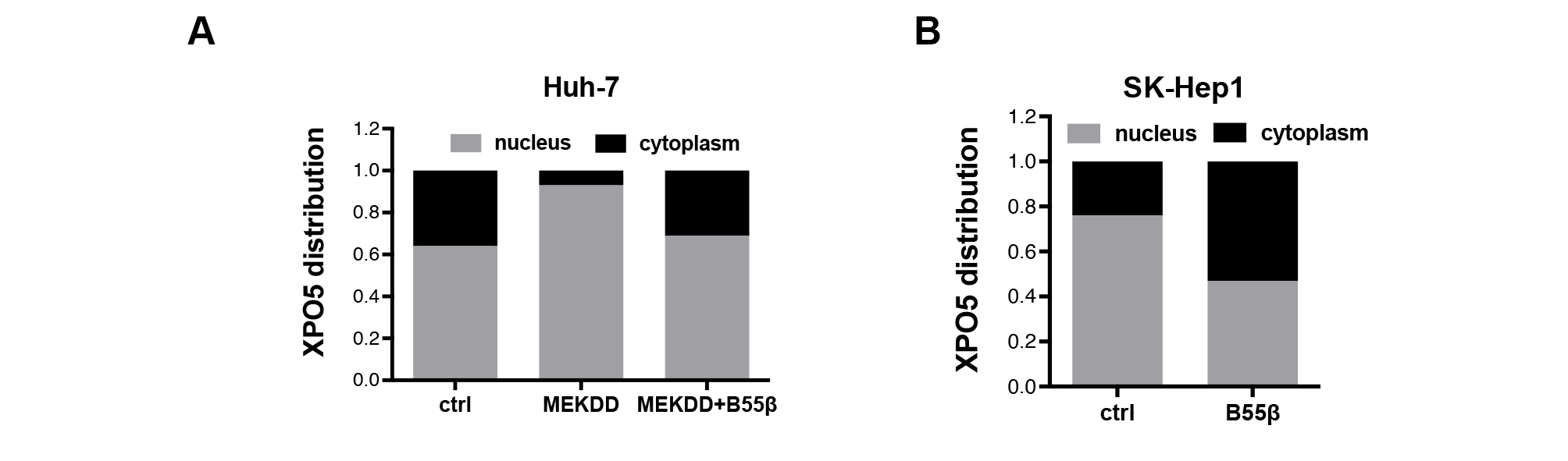
**

**SUPPLEMENTARY FIGURE 2** Fluorescence intensity of XPO5 protein in Huh7 cells (A) and SK-Hep1 cells(B) was determined via ImageJ software. The percentage of XPO5 in the nucleus and the cytoplasm was indicated.
